# PP1 initiates the dephosphorylation of MASTL, triggering mitotic exit and bistability in human cells

**DOI:** 10.1242/jcs.179754

**Published:** 2016-04-01

**Authors:** Samuel Rogers, Dirk Fey, Rachael A. McCloy, Benjamin L. Parker, Nicholas J. Mitchell, Richard J. Payne, Roger J. Daly, David E. James, C. Elizabeth Caldon, D. Neil Watkins, David R. Croucher, Andrew Burgess

**Affiliations:** 1 The Kinghorn Cancer Centre, Garvan Institute of Medical Research, Darlinghurst, New South Wales 2010, Australia; 2 Systems Biology Ireland, University College Dublin, Dublin 4, Ireland; 3 The Charles Perkins Centre, School of Molecular Bioscience and Sydney Medical School, The University of Sydney, Sydney, New South Wales 2006, Australia; 4 School of Chemistry, The University of Sydney, Sydney 2006, New South Wales, Australia; 5 Department of Biochemistry and Molecular Biology, School of Biomedical Sciences Monash University, Clayton, Victoria 3800, Australia; 6 St. Vincent's Clinical School, Faculty of Medicine, UNSW, Darlinghurst 2010, New South Wales, Australia; 7 Department of Thoracic Medicine, St Vincent's Hospital, Darlinghurst, New South Wales 2010, Australia

**Keywords:** Mitotic exit, Greatwall, MASTL, PP1, PP2A, Cdk1, Kinase, Phosphatase, Bistable switch

## Abstract

Entry into mitosis is driven by the phosphorylation of thousands of substrates, under the master control of Cdk1. During entry into mitosis, Cdk1, in collaboration with MASTL kinase, represses the activity of the major mitotic protein phosphatases, PP1 and PP2A, thereby ensuring mitotic substrates remain phosphorylated. For cells to complete and exit mitosis, these phosphorylation events must be removed, and hence, phosphatase activity must be reactivated. This reactivation of phosphatase activity presumably requires the inhibition of MASTL; however, it is not currently understood what deactivates MASTL and how this is achieved. In this study, we identified that PP1 is associated with, and capable of partially dephosphorylating and deactivating, MASTL during mitotic exit. Using mathematical modelling, we were able to confirm that deactivation of MASTL is essential for mitotic exit. Furthermore, small decreases in Cdk1 activity during metaphase are sufficient to initiate the reactivation of PP1, which in turn partially deactivates MASTL to release inhibition of PP2A and, hence, create a feedback loop. This feedback loop drives complete deactivation of MASTL, ensuring a strong switch-like activation of phosphatase activity during mitotic exit.

## Abstract (precis)

**Highlighted Article:** Our results show that loss of Cdk1 activity allows PP1 to partially dephosphorylate MASTL, activating PP2A, which completes the dephosphorylation and deactivation of MASTL, thereby promoting mitotic exit.

## INTRODUCTION

The phosphorylation of proteins by cyclin-dependent kinase 1 (Cdk1) is essential for correct entry into, and progression through, mitosis (Lindqvist et al., 2009). Cdk1 substrates directly and indirectly ensure that the DNA is correctly compacted into chromosomes, and positioned at the centre of the cell through bi-polar attachment to the mitotic spindle. This process is monitored by the spindle assembly checkpoint (SAC), which ensures that Cdk1 remains active until all chromosomes are correctly attached and aligned. Once this is achieved, the SAC becomes satisfied, releasing inhibition of the E3 ubiquitin ligase anaphase-promoting complex comprising the cdc20 subunit (APC^cdc20^), which targets cyclin B1 for destruction by the proteasome, inactivating Cdk1 (Wolf et al., 2006). To exit mitosis, phosphatases reverse the Cdk1-dependent phosphorylation events. Notably, phosphatases are inhibited by Cdk1 activity, consequently, inactivating Cdk1 creates a negative-feedback loop that enhances phosphatase activity, locking the system into irreversible mitotic exit (Yang and Ferrell, 2013). Differentially oscillating activities of Cdk1 and phosphatase enzymes create a two-state system, which comprises interphase and mitosis, respectively (Medema and Lindqvist, 2011). This bistability has been extensively modelled mathematically, especially with regards to the role of Cdk1 and to protein degradation during mitotic exit (López-Avilés et al., 2009; Rattani et al., 2014; Tóth et al., 2007). Consequently, how regulation of Cdk1 activity ensures the irreversibility of mitotic exit is now well established.

Recent studies have focussed on how regulating phosphatases impacts on bistability during mitosis. In yeast, Cdc14 is the primary phosphatase responsible for counter-balancing Cdk1 activity (Bouchoux and Uhlmann, 2011); however, in human cells, it does not appear to play a central role (Mocciaro and Schiebel, 2010). Consequently, in higher eukaryotes, it is unclear which phosphatase(s) regulate mitotic exit. In cycling *Xenopus* extracts, depleting protein phosphatase-1 (PP1) prevents the dephosphorylation of mitotic substrates (Wu et al., 2009), whereas Cdk1-mediated phosphorylation on residue Thr320 of PP1 (which is equivalent to residues Thr316 and Thr311 in PP1β and PP1γ, respectively; and is hereafter referred to as Thr320)' inhibits its activity (Kwon et al., 1997). However, PP2A combined with the B55 subunit (PP2A-B55) has also been proposed as the major phosphatase complex responsible for counterbalancing Cdk1 activity during mitotic exit in human (B55α; PPP2R2A) and *Xenopus* (P55δ; PPP2R2D) systems (Schmitz et al., 2010; Mochida et al., 2009). PP2A-B55 must be inhibited during mitotic entry to ensure that Cdk1 substrates remain phosphorylated during mitosis, and it must be subsequently reactivated upon exit. This mitotic inhibition of PP2A-B55 is under the control of microtubule-associated serine-threonine-like kinase (MASTL) (Burgess et al., 2010; Vigneron et al., 2009). MASTL, originally identified in *Drosophila* as Greatwall (Gwl) (Bettencourt-Dias et al., 2004), is phosphorylated (most probably by Cdk1) on several key residues (Thr194, Thr207, S213 and Thr741), followed by auto-phosphorylation on Ser875 (Blake-Hodek et al., 2012). Active MASTL then phosphorylates two homologous heat-stable proteins – α-endosulfine (ENSA) (Ser67) and Arpp19 (Ser62) (Gharbi-Ayachi et al., 2010; Mochida et al., 2010) – which then bind to the active site of PP2A-B55, acting as an ‘unfair’ competitive inhibitor (Williams et al., 2014). To exit mitosis, Cdk1 substrates must be dephosphorylated; presumably, this requires the deactivation of MASTL, releasing ENSA-mediated repression of PP2A-B55 activity. Interestingly, PP2A-B55 has recently been proposed to dephosphorylate MASTL during mitotic exit (Hégarat et al., 2014), however, because PP2A is inhibited by MASTL, an external trigger is likely to be required to initiate the deactivation of MASTL to kick-start PP2A activity. Here, we demonstrate that PP1 is associated with MASTL during mitotic exit and is capable of dephosphorylating MASTL, correlating with its deactivation. Mathematical modelling showed that PP1 is required for triggering the initial dephosphorylation of MASTL, releasing PP2A inhibition, which completes MASTL and Cdk1 substrate dephosphorylation. In summary, our data provide a unifying theory where both PP1 and PP2A are required for efficient deactivation of MASTL, thereby establishing a bistable switch that drives mitotic exit.

## RESULTS

### Biochemical modelling of mitotic exit in human cells

**Fig. 1.**
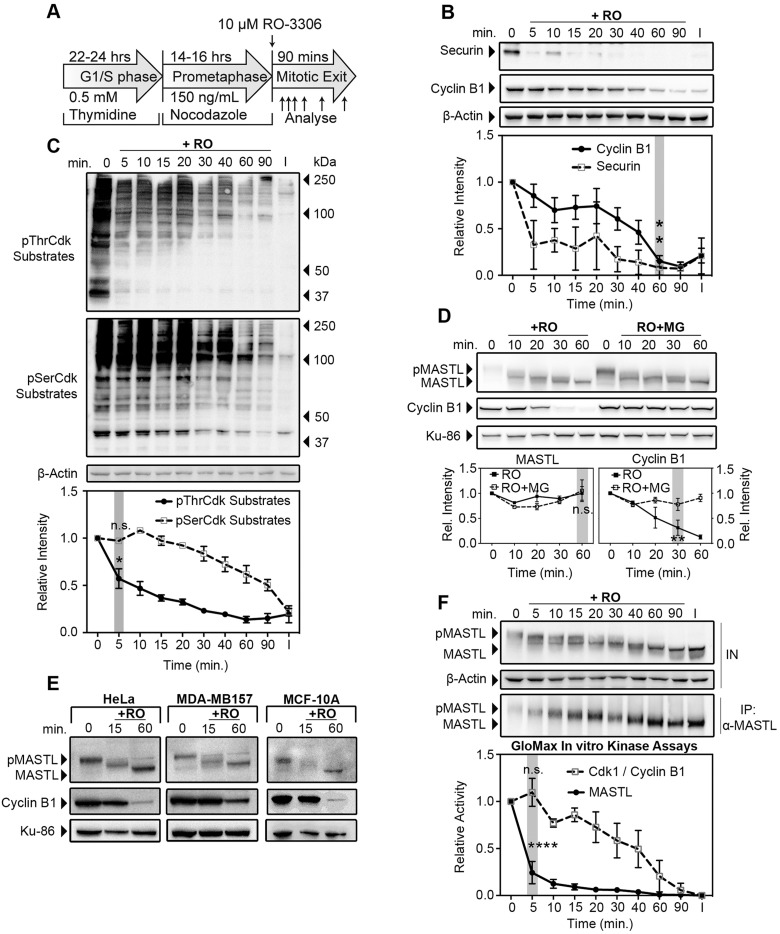
**Mitotic exit within human cells is accompanied by rapid dephosphorylation and deactivation of MASTL.** (A) Schematic of the method used to generate synchronised mitotic exit in human cells. (B,C) HeLa cells synchronised as shown in A were lysed and analysed by western blotting for securin, cyclin B1, pThrCdk substrates, pSerCdk substrates and β-actin (loading). Quantification was performed, and the intensity is expressed relative to the protein level at 0 min. Mean+s.e.m. for *n=*3 values, (one-way ANOVA). I, interphase; n.s., not significant; RO, RO3306. (D) HeLa cells synchronised as per A were treated with 25 μM of the proteasome inhibitor MG-132 (RO+MG) or without (+RO) for 15 min prior to RO3306 addition. Lysates were blotted for MASTL, where phosphorylated MASTL (pMASTL) and dephosphorylated MASTL (MASTL) are indicated by band shift. Cyclin B1 and Ku-86 (loading, also known as XRCC5) were also probed. Quantification was performed, and the intensity is expressed relative to the protein level at 0 min. Mean+s.e.m. for *n*=3 (two-way ANOVA). (E) HeLa (cervical), MDA-MB-157 (breast) and MCF-10A (breast) epithelial cells were treated as shown in A. Lysates were immunoblotted for MASTL, cyclin B1 and Ku-86 (loading). (F) HeLa cells were synchronised as described in A. Cyclin B1 and MASTL were immunoprecipitated (IP) from clear lysates (IN) and then immunoblotted, or assayed using a GloMax Luminescence Kinase Assay kit. Quantification was performed, and MASTL and Cdk1 activity are expressed relative to that at 0 min, corrected to interphase. Mean+s.e.m., *n*=3 (one-way ANOVA). Grey shading indicates significant points; *P<0.05; **P <0.01; ****P <0.0001; n.s., not significant. Cdk1/cyclin B1, Cdk1–cyclin-B1 complex.

To analyse how MASTL is deactivated during mitotic exit, we utilised highly enriched cultures of mitotic human (HeLa) cells, similar to those we and others have used previously (Cundell et al., 2013; Hégarat et al., 2014; McCloy et al., 2014). Briefly, thymidine-synchronised cells were released into nocodazole, and the culture was enriched for prometaphase cells through gentle mitotic shake-off. The Cdk1 inhibitor RO3306 was then added to induce synchronised mitotic exit (Fig. 1A). To validate the synchronised mitotic exit in our model, the APC^cdc20^ substrates securin and cyclin B1 were analysed by western blotting. Securin was rapidly degraded within 5 min, whereas cyclin B1 was slowly degraded throughout the timecourse, reaching interphase levels at approximately 60–90 min post Cdk1 inhibition, indicating that cells had completed mitotic exit by this time (Fig. 1B). Dephosphorylation of mitotic Cdk1 substrates was analysed using phosphorylation-specific antibodies for proline-directed phosphorylated threonine (pThrCdk) and phosphorylated serine (pSerCdk) sites. Significant dephosphorylation of pThrCdk sites was observed within 5 min of RO3306 addition, whereas dephosphorylation of pSerCdk sites occurred with slower linear-like kinetics (Fig. 1C), similar to cyclin B1 degradation (Fig. 1B). This preferential dephosphorylation of pThrCdk substrates mirrors our previous reports on the differential dephosphorylation patterns that occur during mitotic exit (McCloy et al., 2015). Taken together, these results indicate that our system is capable of modelling and temporally separating the early events of mitotic exit, such as chromosome segregation (securin degradation) and the preferential dephosphorylation of pThrCdk substrates, from later events, such as chromosome decondensation and dephosphorylation of pSerCdk substrates.

Previous reports indicate that MASTL activity is primarily regulated through phosphorylation (Burgess et al., 2010); however, degradation of the K71M mutant of MASTL has been reported in *Xenopus* oocytes (Yamamoto et al., 2011), and HSP90 depletion destabilises MASTL (Yamamoto et al., 2014). Therefore, to determine whether degradation contributes to the regulation of MASTL during mitotic exit, total protein levels were quantified either in the presence or absence of the proteasome inhibitor MG132. Treatment with MG132 successfully blocked cyclin B1 degradation for the length of the timecourse (Fig. 1D); however, no significant change in MASTL levels in the presence or absence of MG132 were observed, indicating that degradation does not play a role in regulating MASTL during mitotic exit (Fig. 1D). In contrast, potential dephosphorylation of MASTL, as indicated by a band shift, was noticeable within 10 min, independent of proteasome inhibition. This dephosphorylation of MASTL was also observed in MCF10A and MDA-MB-157 human cell lines (Fig. 1E), indicating that dephosphorylation is likely to be the primary mode of regulating MASTL during mitotic exit in human cells.

Therefore, we next analysed the kinetics of MASTL dephosphorylation and kinase activity. Within 5 min of triggering mitotic exit, a decrease in the mobility of MASTL was observed, which continued step-wise until 90 min, when migration of the protein matched that in interphase cells, indicating complete dephosphorylation (Fig. 1F). Surprisingly, despite this step-wise dephosphorylation, significant loss of kinase activity, as determined by *in vitro* kinase assays, was observed within 5 min of triggering mitotic exit (Fig. 1F). In contrast, Cdk1–cyclin-B1 *in vitro* kinase activity showed a slow linear decline throughout the timecourse (Fig. 1F), which correlated with the degradation of cyclin B1 (Fig. 1B). Taken together, these results suggest that a subset of key phosphorylation sites within MASTL are initially dephosphorylated, causing a small increase in mobility, as detected by observing a band shift, and a significant decrease in kinase activity.

### Analysis of MASTL phosphorylation sites during mitotic exit

**Fig. 2.**
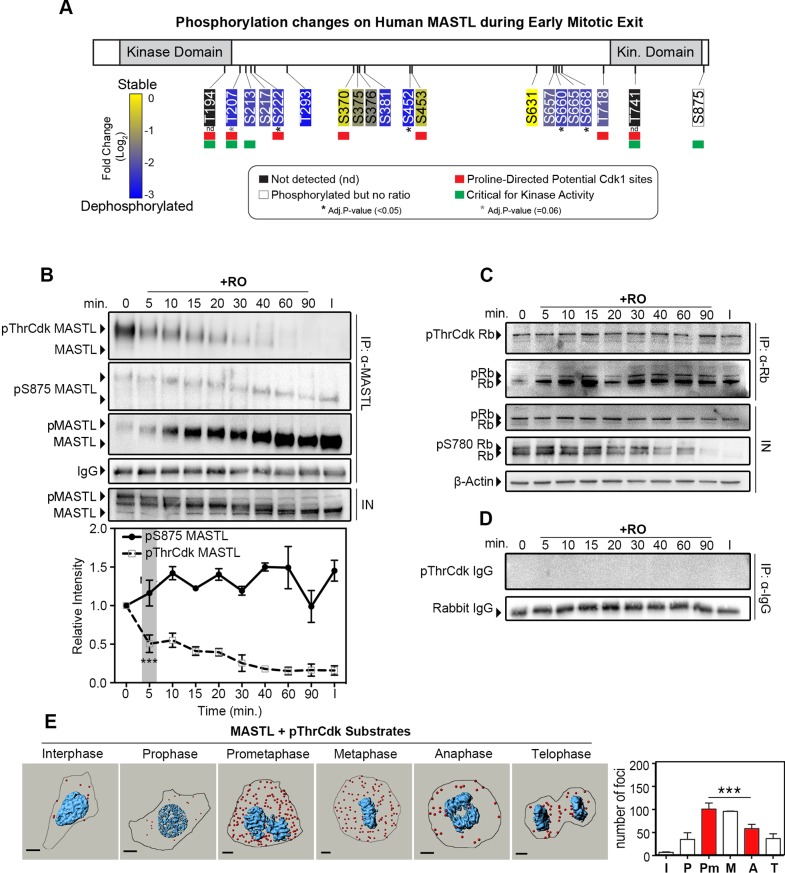
**Cdk1 phosphorylation at threonine residues on MASTL are removed during early mitotic exit.** (A) Summary of MASTL phosphorylation changes during early mitotic exit, as determined by SILAC mass spectrometry analysis. Sites crucial for kinase activity (green), proline-directed potential Cdk1 phosphorylation sites (red) and significantly dephosphorylated sites (*) are shown. Kin., kinase. (B) MASTL was immunoprecipitated (α-MASTL) from extracts as described in Fig. 1B, immunoblotted for total MASTL levels, pThrCdk substrates (pThrCdk MASTL) and MASTL that had been phosphorylated on Ser875 (pS875 MASTL). Quantification was performed, and the intensity is expressed relative to the protein level at 0 min, normalised to IgG loading. *n=*3; ***P<0.001; (one-way ANOVA). (C) Similar to the experiment shown in B, retinoblastoma protein (Rb) was immunoprecipitated (IP: α-Rb), and precipitates blotted for total Rb or pThrCdk substrates (pThrCdk Rb); total lysates were blotted for Rb phosphorylated on Ser780 (pS780 Rb) and β-actin (loading). (D) IgG precipitation (α-IgG) controls corresponding to B and C. (E) PLA using primary antibody pairs against MASTL and pThrCdk substrates. Red dots, interaction foci; blue, nuclei. Data are mean+s.e.m., for a minimum of five cells, *n*=2. Results in prometaphase and anaphase cells were compared (red bars); ***P<0.001 (two-way ANOVA). Scale bars: 10 μm. I, interphase; P, prophase; Pm, prometaphase; M, metaphase; A, anaphase; T, telophase.

Human MASTL has 50 reported phosphorylation sites in the PhosphoSitePlus database (http://www.phosphosite.org/); however, in *Xenopus*, only Thr194, Thr207, Ser213, Thr741 and Ser885 appear to be crucial for kinase activity (Blake-Hodek et al., 2012). To analyse the dephosphorylation of human MASTL during mitotic exit, we mined our recent publication of the global phosphoproteomic mapping of early mitotic exit for MASTL phosphosites (McCloy et al., 2015). This large dataset identified 18 mitotic phosphorylation sites on MASTL, and all exhibited quantitative log_2_ scores, with the exception of Ser875, which owing to the cleavage site of trypsin, did not contain any heavy isotope residues after stable isotope labelling (SILAC) (Fig. 2A). The ratios for the majority of sites showed a fourfold decrease (log_2_ scores <−2, blue), indicating that they were rapidly dephosphorylated during early mitotic exit, with SILAC ratios for Ser222, Ser452, Ser660 and Ser668 all statistically significant (adjusted *P*-values <0.05). The Thr207 site was also rapidly dephosphorylated; however, SILAC ratios were only found in two of the three biological replicates, limiting its significance (*P*-value <0.06).

To analyse the dephosphorylation on MASTL in greater detail, we immunoprecipitated endogenous MASTL using a polyclonal antibody raised against the full-length protein from synchronised mitotic extracts (Fig. 2B). These were then probed with a phosphorylation-specific antibody against the Ser875 auto-phosphorylation site in MASTL (Vigneron et al., 2011). Surprisingly, phosphorylation on Ser875 remained stable throughout mitotic exit, suggesting that this site, although crucial for activity, was not involved in regulating the deactivation of MASTL in our system (Fig. 2B). We therefore turned our attention to the other sites in MASTL that are crucial for activity (Thr194, Thr207, Ser213 and Thr741). Unfortunately, we were unable to produce phosphorylation-specific antibodies for these sites. However, all the threonine sites are proline directed and, therefore, potentially phosphorylated by Cdk1. In support of this notion, both the Thr194 and Thr207 sites can be phosphorylated by Cdk1 *in vitro* (Blake-Hodek et al., 2012). Given this information, we reasoned that the antibody recognising pThrCdk substrates could serve as a surrogate marker for these sites. Probing MASTL immunoprecipitations with the antibody recognising pThrCdk sites revealed a rapid (∼50%) reduction in signal within 5 min of triggering mitotic exit, with complete loss by 60 min (Fig. 2B). In contrast, precipitation of the retinoblastoma protein (Rb; also known as RB1), a substrate we have previously identified to be stably phosphorylated at pThrCdk sites during mitotic exit (McCloy et al., 2015), showed consistent levels of staining of pThrCdk residues throughout the timecourse. Notably, phosphorylation of the Ser780 site on Rb was gradually removed during mitosis (Fig. 2C). Control immunoprecipitations using an antibody against rabbit IgG did not show any cross-reactive staining for pThrCdk sites (Fig. 2D). Taken together, these results suggest that the antibody recognising pThrCdk substrates is able to specifically identify phosphorylated sites within MASTL.

To further validate these findings under normal mitotic exit conditions, we utilised *in situ* proximity ligation assays (PLAs), which detect the colocalisation of two proteins within 40 nm (Rogers et al., 2015a). Negative control PLA assays using antibodies recognising pThrCdk substrates or MASTL in combination with IgG showed very few interactions, consistent with background levels (Fig. S1A,B). Combination of antibodies recognising MASTL and pThrCdk substrates showed a significant increase over background levels, with the number of interactions increasing as cells entered mitosis, peaking during prometaphase and then declining significantly as cells entered anaphase (Fig. 2E). This dephosphorylation trend paralleled the loss observed by western blotting MASTL immunoprecipitations (Fig. 2B) and correlated with the kinetics of deactivation of kinase activity (Fig. 1F). Hence, the fact that MASTL dephosphorylation occurred concurrently with kinase inactivation implies that these events could be correlated.

### Identification of the phosphatase responsible for deactivating MASTL during mitotic exit

**Fig. 3.**
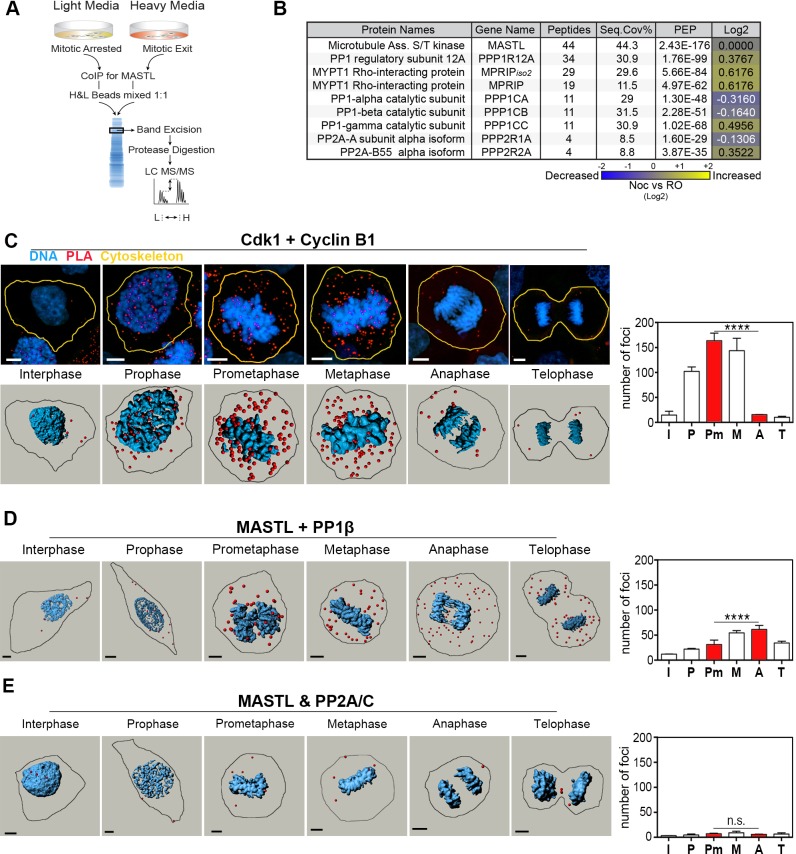
**MASTL binds to PP1 during mitotic exit.** (A) Schematic of SILAC labelling, co-immunoprecipitations and LC-MS/MS analysis of endogenous MASTL. (B) Summary table of MaxQuant analysis of the results obtained using the protocol outlined in A showing the phosphatases identified as being associated with MASTL, posterior error probability (PEP), and log_2_ heavy:light ratios (Log2). Seq. cov, sequence coverage. Noc, nocodazole; RO, RO3306. (C–E) Representative maximum projections (C only) and rendered 3D images of the PLA assay using primary antibody pairs against (C) Cdk1 and cyclin B1 (control), (D) MASTL and PP1β, and (E) MASTL and the catalytic subunit of PP2A, PP2A/C (PPP2CA). Individual PLA dots (red) were quantified using the Imaris dot counter across interphase (I), prophase (P), prometaphase (Pm), metaphase (M), anaphase (A) and telophase (T). The number of foci in prometaphase and anaphase cells were compared (red bars) for *n*>5 cells across two replicates. Blue, nuclei. ****P<0.00001; n.s., not significant (two-way ANOVA). Data are mean+s.e.m. Scale bars: 10 μm. n.s., not significant.

The above results indicate that MASTL activity is regulated by rapid dephosphorylation on key sites during early mitotic exit. To identify the phosphatase responsible for triggering this initial partial dephosphorylation of MASTL, we precipitated MASTL from cells that had been subjected to stable isotope labelling using amino acids in cell culture (SILAC) and then analysed bound proteins by performing quantitative mass spectrometry. Briefly, cells were cultured for 6–7 doublings in the presence of ‘heavy’ or ‘light’ amino acids, as described previously (McCloy et al., 2015). Cells were then synchronised as per Fig. 1A, where ‘light’ samples remained arrested in prometaphase and ‘heavy’ cultures were treated with RO3306 for 20 min to induce mitotic exit. Endogenous MASTL was immunoprecipitated from both cultures, mixed 1:1, separated by gel electrophoresis, digested in-gel and analysed by performing liquid chromatography tandem mass spectrometry (LC-MS/MS) analysis (Fig. 3A). Importantly, MASTL was highly enriched, with good sequence coverage (44.3%) and significant peptide identification confidence (posterior error probability 2.43×10^−176^), providing an internal positive control (Fig. 3B). After filtering for contaminants, a total of 753 unique proteins were identified and quantified by performing mass spectrometry allowing for a 1% false discovery rate (Table S1). Analysis of this dataset revealed eight phosphatase proteins that could potentially interact with MASTL during mitotic exit (Fig. 3B). The list contained the scaffolding A (also known as PPP2R1A) and regulatory B55α subunit of PP2A (PPP2R2A), the regulatory subunit myosin phosphatase MYPT1 (PPP1R12A) and myosin phosphatase Rho-interacting protein (MPRIP), along with the catalytic α, β and γ subunits of PP1 (also known as PPP1CA, PPP1CB and PPP1CC, respectively). Although all three PP1 isoforms were ‘detected’, the majority (11 out of 14) of peptides identified were not isoform specific. The positive SILAC ratios for PP1γ, MYPT1, MPRIP and PP2A-B55 suggest increased association with MASTL during mitotic exit (log_2_ ∼0.3 to 0.6), whereas PP1α and PP1β association with MASTL is slightly reduced. However, these values were all well below the standard twofold-change threshold (log_2_ > 1) normally required for relevance. In summary, the higher confidence identification of multiple PP1-family members suggests that PP1 is the potential MASTL phosphatase.

To confirm the above interactions, additional co-immunoprecipitation and western blot analyses were performed. Immunoprecipitation of endogenous MASTL from prometaphase and early mitotic exit samples showed that there was a strong interaction with the catalytic PP1β subunit, which notably increased in the early mitotic exit (+RO) sample (Fig. S1C, IP1). Further depletion of MASTL from the same extracts failed to recapitulate any noticeable association with PP1β (Fig. S1C, IP2). In addition, MASTL was detected, albeit weakly, in the reverse PP1β immunoprecipitations (Fig. S1D). However, depletion of extracts with a control rabbit IgG antibody was also able to weakly co-precipitate PP1β during the mitotic exit sample (Fig. S1C). The presence of IgG prevented the analysis of the PP2A scaffolding A and B55 subunits in MASTL immunoprecipitations; therefore, we probed for the catalytic subunit of PP2A. However, no association was observed, suggesting that PP2A is not strongly associated with MASTL during early mitotic exit events. Taken together, although MASTL and PP1β appear to be associated during mitotic exit, the non-specificity observed with IgG prevents us from concluding this conclusively.

Therefore, to overcome issues associated with non-specific IgG precipitation of PP1, and to validate the interactions under normal mitotic progression, we utilised PLA assays. To ensure specificity of the antibodies used for PLA, small interfering (si)RNA knockdown of MASTL, PP1β (PPP1CB), the catalytic subunit of PP2A (PPP2CA) and the B55 subunit of PP2A (PPP2R2A) were performed, followed by immunofluorescence analysis. Knockdown of each protein clearly reduced the signal observed in immunofluorescence analysis compared to that observed with the non-targeting controls, indicating that the antibodies specifically recognised their target antigens (Fig. S2A–F). As a positive control for PLA, the proximity of cyclin B1 and Cdk1 was analysed. The number of interactions between cyclin B1 and Cdk1 increased as cells entered mitosis, peaking at prometaphase, before significantly declining as cells entered anaphase and telophase, corresponding with the reported cyclic production and destruction of cyclin B1 in HeLa cells (Fig. 3C) (Chang et al., 2003). An additional control using MASTL mouse (Mo) combined with MASTL rabbit (Rb) antibodies was also performed. The number of interactions between MASTL(Mo) and MASTL(Rb) decreased significantly during prometaphase compared to interphase, and then rose again as cells exited mitosis (Fig. S3A). This change is likely to be due to the reduced ability of the antibodies to completely detect phosphorylated forms of MASTL. Finally, no significant association between IgG and MASTL, and between IgG and PP1β were observed (Figs S1A and S3B), indicating that the non-specific interaction between IgG and PP1 was restricted to the immunoprecipitation analyses. Importantly, PP1β–MASTL PLA assays showed positive interactions that increased significantly as cells progressed from interphase into mitosis, peaking at anaphase, before decreasing in telophase (Fig. 3D). The increase observed during anaphase, although significant, could be due to better antigen retrieval, as noted by the corresponding increase in MASTL(Mo)–MASTL(Rb) interactions (Fig. S3A). In contrast, no significant associations between the catalytic or the B55 subunit of PP2A and MASTL were observed by performing PLA (Fig. 3E; Fig. S3C), indicating that PP2A-B55 is not closely associated with MASTL during mitosis. Taken together, these data indicate that PP1 is associated with MASTL during the early phases of mitotic exit and, hence, is potentially capable of triggering the dephosphorylation of MASTL.

### *In vitro* and *in vivo* dephosphorylation of MASTL by PP1

**Fig. 4.**
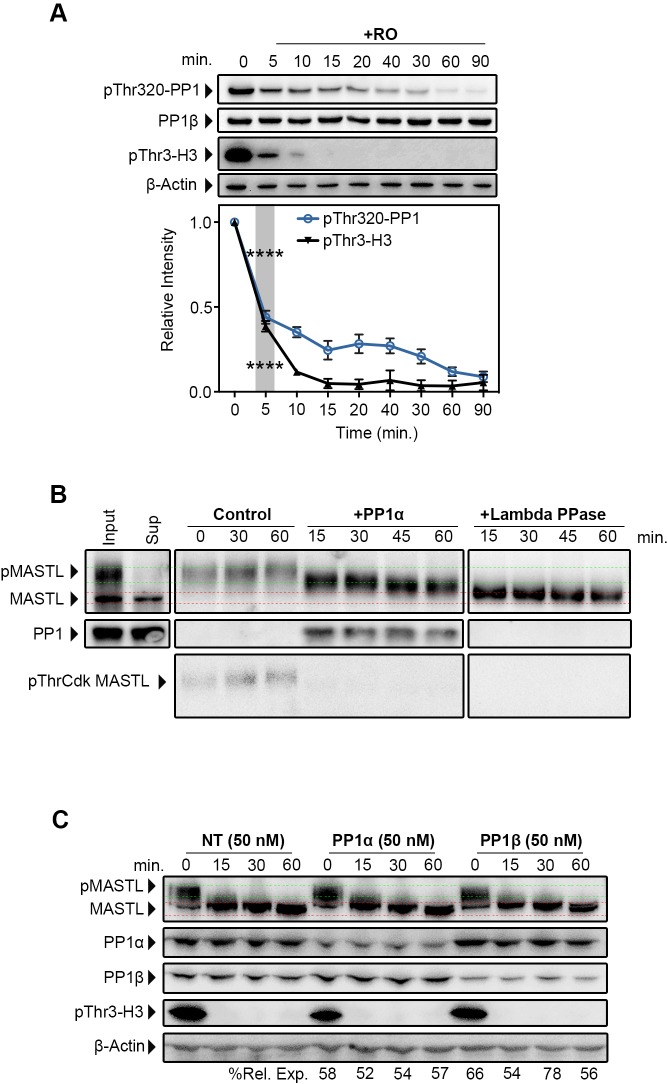
**PP1 is rapidly activated during mitotic exit and capable of partially dephosphorylating MASTL *in vitro*.** (A) Lysates that had been treated as per Fig. 1A were blotted for PP1 that had been phosphorylated on Thr320 (pThr320-PP1), total PP1β, the PP1 substrate histone H3 phosphorylated on Thr3 (pThr3-H3) and β-actin (loading). Quantification was performed, and the intensity is expressed relative to the protein level at 0 min. Mean+s.e.m. for *n*=3. ****P<0.001 (one-way ANOVA). RO, RO3306. (B) MASTL immunoprecipitated from mitotic HeLa extracts, treated with okadaic acid and incubated with recombinant PP1α or λ phosphatase (Lambda PPase) or without (control) for the indicated timepoints. Samples were analysed by western blotting. Phosphorylation of MASTL was assessed by band shift and phosphorylation of pThrCdk sites. (C) HeLa cells that had been transfected with 50 nM of siRNA against PP1α, PP1β or a non-targeting control (NT) for 24 h. Cells were then treated as described in Fig. 1A. Dephosphorylation of MASTL was analysed by band shift, and PP1 activity was measured by pThr3-H3 dephosphorylation. Values indicate the percentage of PP1α and PP1β remaining after knockdown relative to control (%Rel. Exp.).

The above data suggest that PP1 has the potential to deactivate MASTL, possibly by dephosphorylating crucial Thr194, Thr207 and Thr741 pThrCdk sites during early mitotic exit (Fig. 2A). For this model to hold true, it would require rapid reactivation of PP1 during early mitotic exit, to allow subsequent dephosphorylation of MASTL. To test this, we analysed PP1 auto-dephosphorylation of its inhibitory Thr320 site (Wu et al., 2009) in our synchronised mitotic exit system. Significant loss of Thr320 was observed within 5 min of triggering mitotic exit, with complete dephosphorylation of Thr320 occurring within 60 min (Fig. 4A). Accordingly, rapid dephosphorylation of histone H3 on Thr3, a known PP1 substrate (Qian et al., 2011), was also observed within 5 min (Fig. 4A). Taken together, these data indicate that PP1 is activated rapidly upon triggering of mitotic exit with RO3306, with the timing correlating with the dephosphorylation of MASTL.

To examine whether PP1 can dephosphorylate MASTL, we performed *in vitro* phosphatase assays on precipitated MASTL. To ensure maximum MASTL phosphorylation, cells were treated for 60 min with the PP1 and PP2A inhibitor okadaic acid prior to harvesting. Precipitated MASTL was then treated without (control) or with purified PP1α, PP1β or λ phosphatase for 15, 30, 45 or 60 min. MASTL remained phosphorylated in control samples throughout the timecourse (Fig. 4B; Fig. S3D), whereas treatment with λ phosphatase increased MASTL mobility within 15 min and almost completely dephosphorylated MASTL at 60 min (Fig. 4B; Fig. S3E). Similarly, treatment with PP1α (Fig. 4B) and PP1β (Fig. S3D) produced a small but noticeable increase in MASTL mobility within 15 min; however, this only slightly increased over the remainder of the timecourse. This suggests that PP1 specifically targets a subset of the phosphorylated residues on MASTL. In support of this notion, treatment with PP1α almost completely removed phosphorylation of pThrCdk sites within 15 min, whereas phosphorylation of MASTL on Ser875 was only partially reduced after 60 min of treatment with PP1α. Notably, λ phosphatase efficiently removed both pThrCdk site and Ser875 phosphorylation within 15 min, with near-complete dephosphorylation observed after 60 min (Fig. 4B; Fig. S3E).

The above data indicate that PP1α and PP1β can partially dephosphorylate MASTL *in vitro*. To analyse this *in vivo*, HeLa cells were transfected with siRNAs against PP1α or PP1β, and the kinetics of MASTL dephosphorylation was examined using our synchronised mitotic exit model (Fig. 1A). Knockdown of PP1γ, and complete knockdown of PP1α or PP1β caused significant toxicity and inhibited mitotic entry (data not shown). However, sufficient numbers of mitotic cells were obtained with partial (∼20–50%) depletion of PP1α or PP1β. Unfortunately, triggering mitotic exit with 10 µM of RO3306 in cells that had been partially depleted of PP1α or PP1β had no clear effect on MASTL dephosphorylation kinetics. The lack of *in vivo* effects could be due to the potential redundancy between PP1 isoforms, evidenced by both PP1α and PP1β dephosphorylating MASTL *in vitro*. In support, in the sections below, we employed mathematical modelling of mitotic exit to guide our experiments and were subsequently able to observe direct effects of PP1 co-knockdown on MASTL phosphorylation *in vivo*.

### Development of a mathematical model of phosphatase reactivation during mitotic exit

**Fig. 5.**
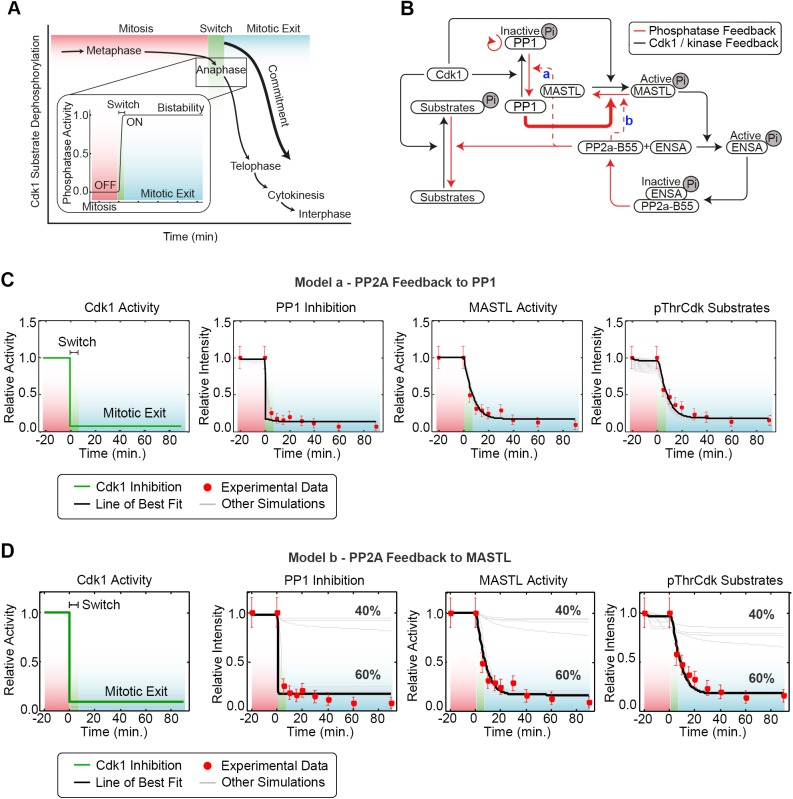
**The bistability of mitotic exit can be modelled using observed experimental parameters.** (A) A model of the bistable phosphatase activity during mitosis, with repression (red shading) during metaphase and activation (blue shading) separated by the bistable switch (green shading). (B) Schematic of the mathematical model, with dephosphorylation of MASTL by PP1 (bold red line) and feedback from PP2A (dotted red line) onto either PP1 (a; model a) or MASTL (b; model b). (C,D) Optimized mathematical model where model a is fitted to training data from 50 independent parameter estimation runs (C). Best fit (black line) depicts 91.1% Cdk1 inhibition. (D) Model b – 60% of the simulations run accurately fit experimental data.

Our data above implicate PP1 as the phosphatase responsible for triggering deactivation of MASTL during mitotic exit. We were unable to confirm this result *in vivo*, most likely because of limitations with sensitivity and toxicity of PP1 knockdown in our mitotic exit system. Therefore, we developed a computational model based on our empirical data to delineate the minimal requirements needed for triggering PP1 dephosphorylation of MASTL and mitotic exit. A central component for mitotic exit is the presence of a bistable switch (green shading, Fig. 5), where the presence of feedback loop(s) triggers the transition between two stable states, mitosis (on, red shading) and interphase (off, blue shading) (Verdugo et al., 2013) (Fig. 5A). The core of our model (Fig. 5B; see Fig. S3F for a reaction kinetic scheme) is based on established models of Cdk1, MASTL, ENSA and PP2A activity. Specifically, our model comprises two feed-forward loops (black lines) from Cdk1, the first suppresses the phosphatase activities of PP1 by phosphorylation of Thr320 (Wu et al., 2009), and the second suppresses PP2A through MASTL phosphorylation of ENSA (Gharbi-Ayachi et al., 2010). Two feedback loops (red lines) were also incorporated – a positive-feedback loop from PP1 to itself and a double negative-feedback loop through inhibition of MASTL and re-activation of PP2A, as indicated by our data (Figs 2 and 3). In the positive loop, PP1 can activate itself through auto-dephosphorylation of its inhibitory Thr320 residue, which is under the control of Cdk1 (Wu et al., 2009). The double negative-feedback loop is initiated by the deactivation of MASTL by PP1, which in turn releases PP2A inhibition (Fig. 5B). To close this double feedback loop, we created two alternative models (Fig. S3F). The first model (Fig. S3Fa, model a) was based on the direct and/or indirect reactivation of PP1 by PP2A through regulation of MYPT1 (Lontay et al., 2005), and on dephosphorylation of inhibitors inhibitor 1 (I-1; also known as PPP1R1A) and Darpp32 (also known as PPP1R1B) (El-Armouche et al., 2006; Wu et al., 2009). In the second model (Fig. S3Fb, model b), PP2A directly feeds back onto MASTL and is responsible for the removal of additional phosphate moieties added by Cdk1 and for the inhibition of MASTL activity (Hégarat et al., 2014). All equations, reaction values and estimated parameter values are listed in Tables S2–S6.

To train these models, we utilised the biological data generated from our highly synchronised mitotic exit system detailed above (Figs 1A–F,2B,4A) – combined with the reported published abundance for each protein in HeLa cells from the MOPED database (https://www.proteinspire.org; Kolker et al., 2012) – and the reported *K*_m_ and *K*_cat_ values for the dephosphorylation of ENSA by PP2A-B55 (Williams et al., 2014). The remaining dephosphorylation rates were uncertain and, thus, estimated using the model in Fig. S3Fa and adaptive simulated annealing, a method for global parameter estimation (Ingber, 1989). To assess the associated uncertainty, a Monte-Carlo-based approach was used where the initial parameters were randomly changed 50 times and the model refitted to the experimental data (Hengl et al., 2007; Maiwald and Timmer, 2008). We then analysed parameter correlations and variability of the simulated predictions, and although the exact parameter values were not uniquely identifiable, their estimates occupied highly structured regions in the parameter space resulting in parameter correlations (Fig. S3G).

For the model in Fig. S3Fa, all 50 estimated models (light grey lines), and in particular the best-fitting model (solid back line), closely matched our experimental data (red dots) for the kinetics of PP1 inhibition (Thr320 dephosphorylation), MASTL kinase activity and the dephosphorylation of global pThrCdk sites – a readout of PP2A reactivation and mitotic exit (Fig. 5C). Using the same parameter estimates, the model shown in Fig. S3Fb was also able to closely match our experimental data in 60% of the simulations (Fig. 5D). This indicates that the differences between the two models are not crucial for explaining these data and, therefore, for simplicity, we choose the model shown in Fig. S3Fa for further analysis.

### Validation of the model

**Fig. 6.**
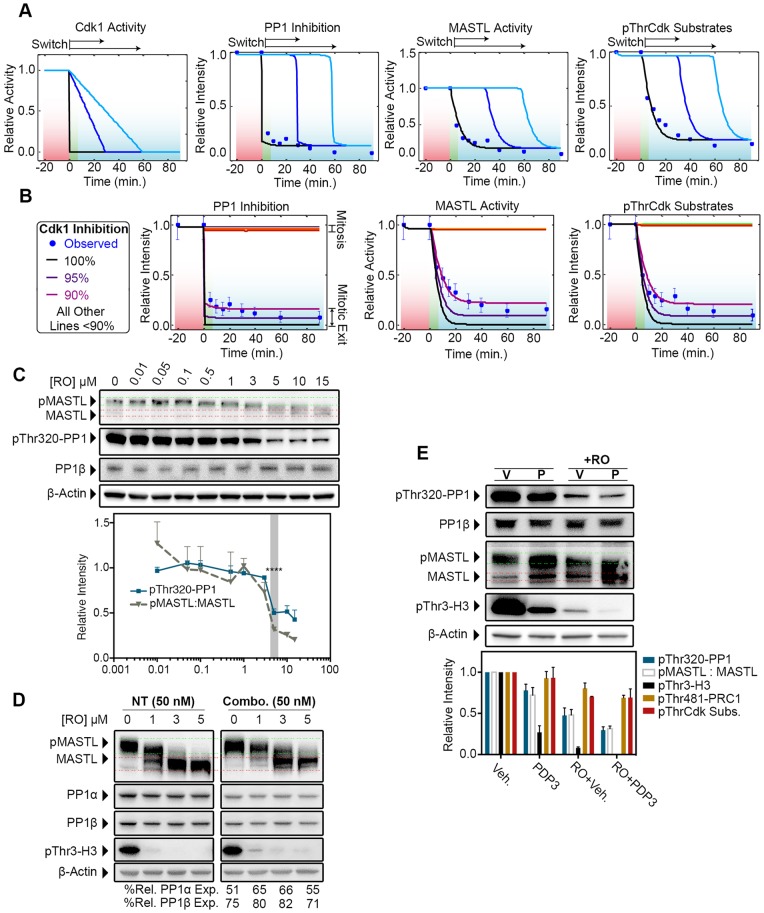
**A threshold of Cdk1 activity and PP1 are required to trigger the mitotic exit switch.** (A) Rate of Cdk1 inhibition from model a was changed to incorporate two different linear decay rates (blue lines), and subsequent effects on PP1 inhibition, MASTL activity and pThrCdk substrate dephosphorylation modelled. (B) Inhibition of Cdk1 was varied from 5 to 100% in 5% increments using model a. (C) Validation of Cdk1 bistability threshold. Whole-cell lysates that had been treated with RO3306 (RO) for 15 min were probed by western blotting for total PP1β, PP1 phosphorylated at Thr320 (pThr320-PP1) and MASTL. Shown are representative blots, and quantification was performed – the intensity is expressed relative to the protein level at time 0 min. Mean+s.e.m., *n*=3. MASTL activity was estimated by quantifying the ratio of phosphorylated MASTL (pMASTL) (green lines) to MASTL (red lines) (pMASTL:MASTL); bistability is highlighted (grey shading). ****P<0.0001 (ordinary one-way ANOVA). (D) HeLa cells that had been co-depleted of PP1α (25 nM) and PP1β (25 nM) (combo) or with non-targeting (NT, control) siRNA were synchronised, as per Fig. 1A, treated with increasing concentrations of RO3306 for 30 min and assessed by western blotting for knockdown efficiency, dephosphorylation of MASTL (band shift) and of PP1 substrate histone H3 (pThr3-H3). Values represent the amount of PP1α and PP1β remaining, relative to that with control treatments (%Rel. Exp.). *n*=2. (E) HeLa cells were synchronised as described in Fig. 1A but treated with the PP1-activating peptide PDP3 (40 μM, P) or vehicle (V, Veh) control for 3 h prior to harvest. Samples were treated with (+RO) or without 3 µM RO3306 for 30 min. Lysates were analysed by western blotting and quantified relative to vehicle (far left lane), for pThr320-PP1, total PP1β, MASTL, pThr3-H3 and β-actin [see Fig. S4B for PRC1 phosphorylated at Thr481 (pThr481-PRC1) and pThrCdk substrates (pThrCdk Subs)]; *n*=2.

One limitation of the above simulations and our biological system is that they relied on instantaneous inhibition of Cdk1. Therefore, our first simulation was to test whether bistable switch-like dynamics would be maintained if Cdk1 activity declined gradually, similar to the normal degradation of cyclin B1. Simulating the model with two different decay rates of Cdk1 activity delayed the onset of mitotic exit (Fig. 6A). Importantly, this delay did not alter the switch-like behaviour with regards to PP1 inhibition, deactivation of MASTL and subsequent dephosphorylation of pThrCdk substrates, which all occurred with similar kinetics to those observed with instantaneous Cdk1 inhibition. This indicates that Cdk1 activity determines the timing of mitotic exit, and once Cdk1 activity drops below a certain threshold, the bistable phosphatase switch is triggered and mitotic exit occurs. Therefore, our method of instantaneous Cdk1 inhibition is a useful method for modelling mitotic exit.

Next, we utilised the model to determine the threshold of Cdk1 activity that triggers the bistable switch and mitotic exit. Surprisingly, the model identified a clear bistable threshold, with 90% or greater inhibition of Cdk1 required to trigger PP1 activation, and MASTL and pThrCdk substrate dephosphorylation (Fig. 6B). To validate this experimentally, increasing doses of RO3306 were added to Nocodazole-arrested cells for 30 min. No significant effect on the dephosphorylation of MASTL (as determined by band shift) was observed below concentrations of 1 µM of RO3306 (Fig. 6C). A small increase in MASTL mobility and dephosphorylation of PP1 on residue Thr320 was observed at 3 µM, which became clear and significant at 5 µM. Notably, a dose of 5 µM has been reported to inhibit up to 90% of cyclin-B–Cdk1 activity (Vassilev et al., 2006), matching the predictions made by our model.

Based on these data, we hypothesised that sub-optimal doses of RO3306 at the switching threshold for Cdk1 might allow subtle changes in MASTL dephosphorylation to be observed with siRNA depletion of PP1. To test this, we repeated the partial knockdown of PP1α and PP1β and then treated cells with 1–5 µM of RO3306 for 30 min. No clear effect was seen when PP1α or PP1β were depleted individually (Fig. S4A); however, partial co-depletion of PP1α and PP1β produced a small but noticeable increase in the slower-migrating form of MASTL at both 1 and 3 µM (Fig. 6D). This corresponded with a slight increase in the amount of histone H3 that had was phosphorylated at Thr3, indicating that PP1 was partially inhibited. Taken together, these data suggest that PP1 is capable of directly regulating the dephosphorylation of MASTL during mitotic exit. Furthermore, there is some redundancy between PP1α and PP1β, which is likely to explain why we failed to observe any effect in single-knockdown experiments.

To further validate PP1 dephosphorylation of MASTL *in vivo*, we employed a novel peptide activator (PDP3) of PP1 (Chatterjee et al., 2012). Treatment of a population of prometaphase-arrested cells with PDP3 induced a small (∼20%) decrease in phosphorylation at Thr320 on PP1. This corresponded with significant dephosphorylation on Thr3 of histone H3, indicating that PP1 is reactivated by the peptide (Fig. 6E). To estimate the level of MASTL dephosphorylation, we compared the ratio between the phosphorylated (Fig. 6E, upper bands, green) and dephosphorylated (Fig. 6E, lower bands, red) migrating bands. The levels of MASTL dephosphorylation closely matched those of dephosphorylation at Thr320 on PP1, with treatment with PDP3 alone inducing a ∼20% increase in MASTL mobility. Interestingly, this partial ∼20% activation of PP1 and inhibition of MASTL did not trigger mitotic exit, with PP2A substrates (pThrCdk sites and PRC1 at Thr481) remaining phosphorylated (Fig. 6E; Fig. S4B). Next, we hypothesised that combination of PDP3 with partial inhibition of Cdk1 just below the bistability threshold might trigger mitotic exit. Addition of 3 µM RO3306 reduced phosphorylation of Thr320 on PP1 by ∼50% that, when combined with PDP3, reduced to ∼75% of control levels and resulted in a near-complete dephosphorylation of histone H3 at Thr3. MASTL dephosphorylation coincided with PP1 reactivation, with treatment with RO3306 increasing mobility by ∼50%, whereas PDP3 in combination with RO3306 reduced phosphorylation by ∼75% (Fig. 6E). Surprisingly, although addition of 3 µM RO3306 did increase the dephosphorylation of pThrCdk sites and PRC1 (at Thr481) more than PDP3 alone, the combination of PDP3 and RO3306 did not significantly increase dephosphorylation further (Fig. 6E; Fig. S4B), indicating that PP2A remained partially inhibited under these conditions. These data were used to update all the parameter estimations in our mathematical model (Fig. S4C). Importantly, introduction of partial (20–40%) activation of PP1 and subsequent MASTL deactivation corresponded with a similar partial dephosphorylation of pThrCdk substrates but did not trigger the bistable mitotic exit switch (Fig. S4D). Furthermore, this revised model (a rev) was still able to accurately model our experimental data based on rapid (90%) Cdk1 inhibition (Fig. S4E). Notably, previous reports in *Xenopus* cell-free extracts have indicated that as little as ∼30% of MASTL activity is sufficient for maintaining phosphorylation of mitotic substrates (Blake-Hodek et al., 2012; Vigneron et al., 2011), which is likely to explain why partial dephosphorylation and deactivation of MASTL by PP1 is insufficient to drive mitotic exit by itself.

### MASTL deactivation is essential for mitotic exit and requires both PP1 and PP2A

**Fig. 7.**
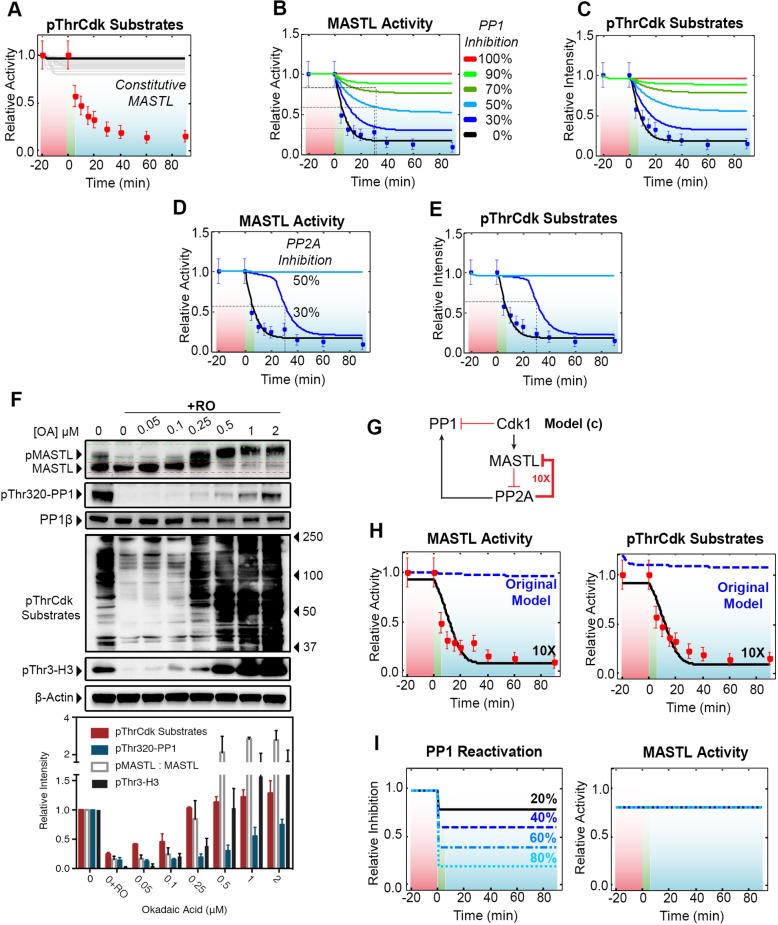
**PP2A-PP1 feedback is crucial for ultrasensitivity and bistability.** (A) Effects of constitutively active MASTL on mitotic exit were simulated 50 times (grey lines) using model a. (B,C) Using model a, the effect of PP1 inhibition on MASTL activity and pThrCdk substrates was simulated. Removal of the PP1 auto-dephosphorylation loop (red line) completely abolished PP1 activity, disrupting bistability. Simulating partial inhibition of PP1 activity from 30–90% (blue and green lines) by reducing PP1 autoactivation (see k2b Fig. S3F) resulted in a corresponding increase in the relative activity of MASTL and pThrCdk substrate phosphorylation. (D,E) Similar to the models in A and B, except for the effects of PP2A inhibition on MASTL and pThrCdk substrate phosphorylation were simulated by reducing k4 (see k4b; Fig. S3F) as measured by MASTL activity and pThrCdk substrates. (F) HeLa cells synchronised as per Fig. 1A were treated for 1 h with increasing doses of the PP1 and PP2A inhibitor okadaic acid (OA) followed by 10 µM RO3306 (RO) for 30 min. Total cell lysates were analysed by western blotting, and the levels of total PP1β, PP1 phosphorylated at Thr320 (pThr320-PP1), histone H3 phosphorylated at Thr3 (pThr3-H3) and pThrCdk substrates, as well as the ratio of phosphorylated MASTL to total MASTL (pMASTL:MASTL), were quantified as a measure of mitotic exit and bistability. *n*=2 repeats. (G) Schematic of the final model c, which lacks the dephosphorylation of MASTL by PP1. (H) In the model a rev (dotted blue line), MASTL dephosphorylation is not triggered and pThrCdk substrates are not dephosphorylated, unless PP2A feedback on MASTL is amplified tenfold (model c, bold red line). (I) In model c, PP1 is activated to varying levels (black and blue lines) in place of Cdk1 inhibition, and the effect on MASTL activity is measured.

An important hypothesis of this work is that MASTL must be deactivated to permit mitotic exit. In support, we have previously shown in *Xenopus* extracts that the mitotic state is lost upon depletion of MASTL, even when Cdk activity is maintained (Vigneron et al., 2009). However, to date, no one has been able to produce a constitutively active form of MASTL with which to validate this hypothesis. Therefore, we introduced a constitutively active form of MASTL into our model. Interestingly, this completely prevented exit and bistability (Fig. 7A). Similarly, complete removal of the auto-dephosphorylation loop on PP1 was sufficient to prevent the bistable switch at the estimated level of Cdk1 inhibitor effectiveness (dCdk1=0.911) (Fig. 7B, red line). To examine the dynamics of PP1-mediated MASTL deactivation in more detail, we simulated the effects on MASTL deactivation upon increasing levels of PP1 inhibition. Increasing inhibition of PP1 from 30 to 70% caused incomplete deactivation of MASTL and dephosphorylation of pThrCdk substrates without significantly affecting the shape of the response (Fig. 7B,C). However, to completely prevent MASTL deactivation and to block mitotic exit, >90% PP1 inhibition was required (Fig. 7A,B, light green line). Further, the response to PP1 inhibition was gradual, resembling a linear rather than an ultrasensitive switch-like curve; hence, there is no pronounced threshold at which mitotic exit is prevented, further explaining why partial siRNA-mediated depletion or reactivation of PP1 with PDP3 only had a small effect on MASTL dephosphorylation. Therefore, we next examined the impact that PP2A has on MASTL deactivation and bistability in our model. In contrast to that of PP1, the PP2A response was highly nonlinear and switch-like. Partial inhibition of PP2A (30%; Fig. 7, dark blue line) significantly delayed the kinetics of mitotic exit, and the bistable switch was completely abolished at 50% inhibition of PP2A (light blue line) (Fig. 7D,E). This non-linear switch-like response indicates that the feedback loop from PP2A to PP1 (and/or MASTL) is crucial for generating the required ultrasensitivity. To validate this prediction, we took advantage of the 10–100-fold differential selectivity of okadaic acid for PP2A over PP1 (Swingle et al., 2007), with doses below 1 µM reported to be specific for PP2A in human cell lines (Favre et al., 1997). A HeLa cell culture that had been enriched for prometaphase cells was exposed to increasing doses of okadaic acid (0–2000 nM) prior to triggering mitotic exit with RO3306. Based on our model, mitotic exit and MASTL dephosphorylation should be rescued when >50% inhibition of PP2A is achieved. In support of this hypothesis, rescue of MASTL and global pThrCdk phosphorylation was observed at 50–100 nM, with near-complete rescue observed at 500 nM of okadaic acid (Fig. 7E). In contrast, a dose of 1–2000 nM was required to rescue the auto-dephosphorylation of PP1 on Thr320 and to significantly increase the levels of phosphorylation of histone H3 on Thr3, indicating substantial PP1 inhibition. Interestingly, these high doses were able to further increase the band shift observed for MASTL, further supporting the notion of a role for PP1 in triggering a partial dephosphorylation of MASTL.

To test the importance of PP1 for MASTL dephosphorylation, we created a final alternative model (model c), where the negative feedback from PP1 to MASTL was removed, but feedback from PP2A to MASTL and PP1 remained (Fig. 7G). Simulations with this alternative model (black lines, Fig. S4F) closely matched the findings of our experimental data (blue dashed lines) for the dephosphorylation of PP1 observed upon inhibition of Cdk1. However, it was unable to model the dephosphorylation of MASTL or pThrCdk substrates, the levels of which remained stable (Fig. 7H, blue dashed lines). Artificially increasing the dephosphorylation rate (parameter k4; Fig. S3F) of MASTL tenfold allowed the alternative model (Fig. 7G) to fit the experimental results for MASTL and pThrCdk dephosphorylation (Fig. 7H, black lines). However, when we tested the model using increasing activation of PP1 (20–80%) instead of Cdk1 inhibition, there was no effect on MASTL dephosphorylation or on pThrCdk substrates (Fig. 7I). Therefore, even under these artificial conditions with highly unstable phosphorylation of MASTL, PP1 is still absolutely required to initiate the dephosphorylation of MASTL, ensuring sufficient reactivation of PP2A, which then triggers the rapid bistable mitotic exit switch.

## DISCUSSION

Our results show that rapid dephosphorylation and deactivation of MASTL is crucial for ensuring bistability during mitotic exit. We propose that PP1 initiates the dephosphorylation of MASTL, relieving the inhibition of PP2A, which then completes the full deactivation of MASTL. The degradation of cyclin B, which begins during metaphase (Gavet and Pines, 2010), initiates mitotic exit because decreasing levels of cyclin B are likely to be sufficient to relieve Cdk1 phosphorylation of Thr320 on PP1, allowing auto-dephosphorylation and activation. Thus, we believe that our model of bistable phosphatase reactivation works in combination with the established bistable models of mitotic exit (López-Avilés et al., 2009; Yang and Ferrell, 2013) and that this model accurately represents the ultrasensitivity and irreversibility of the mitotic exit switch.

Recent reports in fission yeast demonstrate that partial reactivation of PP1 is required for subsequent PP2A-B55 and PP2A-B56 (which comprises the PPP2R5A subunit) reactivation during mitotic exit. Although the mitotic exit roles of PP1 and PP2A in yeast and humans are not entirely conserved, this relay switch appears to be maintained (Grallert et al., 2014). Furthermore, human cells that have been depleted of PP2A-B55 delay during anaphase rather than metaphase, with defects in post-mitotic spindle breakdown and nuclear envelope reassembly (Schmitz et al., 2010). If PP2A was responsible for the initial dephosphorylation of MASTL, then an earlier metaphase arrest similar to that induced by non-degradable cyclin B would be expected (Chang et al., 2003). This interdependence of PP1 and PP2A for bistability in our model also provides an explanation for previous reports that PP2A dephosphorylates MASTL during mitotic exit (Hégarat et al., 2014). In our model, PP2A is part of the ultrasensitive feedback loop regulating PP1 and MASTL, and is needed for the establishment of bistability. Consequently, partial inhibition of PP2A is sufficient to disrupt PP1 reactivation and to block Cdk1 substrate dephosphorylation, thereby maintaining MASTL activity and preventing mitotic exit. This accounts for the ability of the phosphatase inhibitor okadaic acid to rescue MASTL phosphorylation and to block mitotic exit at doses specific for inhibition of PP2A. Additionally, our data show that >90% of PP1 needs to be inhibited to maintain complete phosphorylation of MASTL and pThrCdk substrates. This is likely to explain why complete depletion of PP1 prevents Cdk1 substrate dephosphorylation and blocks mitotic exit in *Xenopus* extracts (Wu et al., 2009). In contrast, inhibitors, such as tautomycin, are unlikely to achieve this level of inhibition in human cells (Favre et al., 1997), explaining why these experiments have failed previously.

The final question remaining is how does PP1 initiate the deactivation of MASTL? We believe that PP1 dephosphorylates key pThrCdk residues (Thr194, Thr207 and Thr741), initiating MASTL deactivation. In support of this idea, mutating these sites to non-phosphorylatable alanine residues reduces MASTL kinase activity by up to 75%, triggering mitotic exit in *Xenopus* extracts (Blake-Hodek et al., 2012). In addition, the amino acids surrounding Thr207 match the TPG motif, which we have previously identified as a highly unstable phosphorylation motif (McCloy et al., 2015). Similarly, dephosphorylation at Thr194 during mitotic exit has also been observed in a similar model of mitotic exit (Hégarat et al., 2014). While preparing this manuscript, Heim et al. demonstrated in *Xenopus* extracts that MASTL is also dephosphorylated by PP1. Using several elegant experiments using a mutant (G41S), which lacks auto-phosphorylation ability, they propose that the Ser883 site (equivalent to Ser875 in human MASTL) is dephosphorylated by PP1 (Heim et al., 2015). However, this mutant, which appears to act as a dominant-negative protein (Vera et al., 2015), was only partially phosphorylated during mitosis, and changes in phosphorylation on Ser883 were not directly assessed. In contrast, we did not observe any significant decrease in phosphorylation during mitotic exit using a phosphorylation-specific antibody against human Ser875, although PP1 could partially dephosphorylate this site *in vitro*. A probable explanation for these differences is that PP1 can target multiple sites on MASTL during early mitotic exit, providing multiple different steady-states of MASTL (Thomson and Gunawardena, 2009). These multiple sites could be targeted by different PP1 isoforms, explaining the redundancy we observed between the α and β isoforms. In addition, PP1γ, which we also identified by performing mass spectrometry, might also be involved in regulating MASTL. Consequently, future studies will be needed to identify the specific phosphosites that are regulated by the various PP1 and PP2A complexes to fully understand how these phosphatases regulate MASTL and, ultimately, mitotic exit in human cells.

In summary, we believe that our model provides an attractive unifying theory where mitotic exit is triggered by an initial loss of Cdk1 activity during late metaphase, allowing PP1 to begin the dephosphorylation of MASTL, thereby releasing PP2A inhibition and triggering the bistable switch that locks cells into the mitotic exit pathway.

## MATERIALS AND METHODS

### Antibodies, chemicals and reagents

All antibodies used are listed in Table S7. Anti-MASTL rabbit polyclonal and phospho-specific antibodies against Ser875 were generated as previously described (Burgess et al., 2010; Vigneron et al., 2011). The following chemicals were used: RO3306 (Axon MedChem), okadaic acid sodium salt (A.G. Scientifix), nocodazole (Sigma-Aldrich), thymidine 2′-deoxycytidine hydrate (Santa Cruz Biotechnology), protease inhibitor cocktail (PIC) (Sigma-Aldrich), adenosine 5′-triphosphate (New England Biolabs), (*S*)-MG132 (Cayman Chemicals), iodoacetamide (Sigma-Aldrich) and TCEP-hydrochloride (ThermoFisher Scientific). PDP3 peptide (Reither et al., 2013) was synthesised using H-Rink Amide ChemMatrix resin at a loading concentration of 0.52 mmol/g. Automated and manual solid-phase peptide synthesis (SPPS), preparative and analytical high-performance liquid chromatography and analytical LC-MS was performed as described previously (Mitchell et al., 2015).

### Cell culture and synchrony

All cell lines were validated with a >90% match at CellBank Australia or American Type Culture Collection using short tandem repeat (STR) profiling, and grown as previously described (Browne et al., 2013). HeLa cells were synchronised as previously described (McCloy et al., 2015). For mitotic exit synchrony, cells were treated with 10 μM RO3306.

### siRNA design and transfection

The Promega T7 Ribomax Express RNAi system was used to produce *in vitro* transcribed siRNAs against coding DNA sequence (CDS) target sequences for MASTL (pool) **647** 5ʹ-GCTCGTTGGGATTTAACAC-3ʹ, **1144** 5ʹ-GGACGCTCTTGTGTAAACC-3ʹ, **1879** 5ʹ-GCTGTACAAGAGAGTAACC-3ʹ; PP1α **180** 5ʹ-GATCTGCGGTGACATACAC-3ʹ; PP1β **619** 5ʹ-GATCCAGATAAGGATGTGC-3ʹ; PP2A catalytic subunit **784** 5ʹ-GCTCCAAACTATTGTTATC-3ʹ; PP2A-B55 **1297** 5ʹ-GTAGCTACTACAAACAATC-3ʹ; non-targeting control (NT) 5ʹ-GGATTGTGCGGTCATTAACTT-3ʹ. Numbers in bold denote the starting nucleotide number of the sequence within the CDS. Transfection of siRNA was performed using Lipofectamine 3000 reagent (Invitrogen), as per the manufacturer's instructions.

### Western blot, immunoprecipitation and phosphatase and kinase assays

Western blots were performed and quantified as previously described (McCloy et al., 2014, 2015). For immunoprecipitations, protein A/G magnetic beads (Pierce) were mixed with antibodies for 1.5–2 h at room temperature before addition to lysates for 2 h at room temperature. For *in vitro* phosphatase assays, HeLa cultures enriched for prometaphase cells were treated with 100 nM okadaic acid for 1 h before harvesting. Cell pellets were lysed in immunoprecipitation lysis buffer and MASTL was immunoprecipitated (250 µg), and beads were washed three times with immunoprecipitation lysis buffer, three times with 20 mM Tris-HCl (pH 8) and resuspended in 50 µl of 1×NEBuffer for protein metallophosphatases supplemented with 1 mM MnCl_2_. MASTL immunoprecipitates were then treated without (Control) or with purified active PP1α (1 unit, NEB, #P0754S), PP1β (0.1 µg, Abcam, #ab128551), or λ phosphatase (200 units, NEB, #P0753S) for the indicated times at 30°C. Reactions were stopped with 2× LDS-PAGE buffer with dithiothreitol and then boiled at 95°C for 5 min. All samples were analysed by western blotting. Co-immunoprecipitations were performed identically, except without okadaic acid. IgG- or primary-antibody-bound beads were mixed with lysates for 16–18 h at 4°C. In some cases, a second round of depletion was performed for 2 h at room temperature. For MASTL kinase assays, mitotic exit and interphase synchronised cells were treated as per the immunoprecipitation protocol. Beads were washed in immunoprecipitation lysis buffer (without NP-40), then kinase reaction buffer (KRB) (20 mM HEPES, 10 mM MgCl_2_, 0.1 mg ml^−1^ BSA, 1 mM DTT). Beads were resuspended in KRB plus 10 μg myelin basic protein and ATP to a final concentration of 400 μM, incubated for 1 h at 37°C and analysed using Kinase GloMax Luminescence kit (Promega) as per the manufacturer's instructions. The values for MASTL activity were normalized to the depletion efficiency of the total MASTL immunoprecipitation.

### Immunofluorescence and proximity ligation assays

Immunofluorescence (McCloy et al., 2014) and PLA assays (Rogers et al., 2015a) were performed as previously described. All coverslips were mounted in ProLong Gold (Life Technologies) and imaged on a Leica DMI6000 SP8 confocal microscope with a 63×1.4 lens at 1024×1024 resolution powered by LAS AF v8.0. PLA dots were counted and annotated using Imaris v8.1, as described previously (Burgess et al., 2012; Rogers et al., 2015a). FIJI-ImageJ v1.50a and Adobe Photoshop CC 2015 were used for image colouring and overlays.

### SILAC labelling and mass spectrometry

HeLa cells were SILAC-labelled, as previously described (McCloy et al., 2015), with immunoprecipitation and peptide purification described as per Di Virgilio et al. (2013). Mass spectrometry, peptide analysis and bioinformatics was performed as described previously (McCloy et al., 2015; Rogers et al., 2015b).
